# 纵隔镜与EBUS-TBNA对肺癌诊断和分期价值的比较研究

**DOI:** 10.3779/j.issn.1009-3419.2013.06.03

**Published:** 2013-06-20

**Authors:** 良 张, 锋 毛, 明辉 蔡, 阳 申屠

**Affiliations:** 200030 上海，上海交通大学附属胸科医院/上海市肺部肿瘤临床医学中心胸外科 Shanghai Chest Hospital, Shanghai Jiaotong University, Department of Thoracic Cancer, Shanghai Lung Tumor Clinical Medical Center, Shanghai 200030, China

**Keywords:** 纵隔镜检查术, 支气管镜超声引导针吸活检术, 肺肿瘤, 纵隔肿物, Mediastinoscopy, Endobronchial ultrasound-guided transbronchial needle aspiration, Lung neoplasms, Mediastinal mass

## Abstract

**背景与目的:**

支气管镜超声引导针吸活检术（endobronchial ultrasound-guided transbronchial needle aspiration, EBUS-TBNA）逐渐普及，但其对肺癌的术前分期和纵隔肿物的诊断价值尚待探讨。本研究拟对比研究EBUS-TBNA和纵隔镜的诊断效能，期望为理性的选择应用提供客观依据。

**方法:**

收集2009年7月-2012年12月纵隔镜检查术患者361例，其中肺癌199例，纵隔肿物162例；EBUS-TBNA患者348例，其中肺癌216例，纵隔肿物132例。比较两种方法的诊断结果和相关指标，分析二者对肺癌术前分期和纵隔肿物诊断的临床应用价值。

**结果:**

以石蜡病理学诊断为金标准，纵隔镜检查术的诊断准确性为98.33%，敏感性98.17%，特异性100%；EBUS-TBNA的准确性为90.80%，敏感性90.00%，特异性100%。两者对肺癌的诊断和分期效能无明显差异（*P* > 0.05），纵隔镜对纵隔肿物的诊断效能较高（*P* < 0.05）。

**结论:**

纵隔镜和EBUS-TBNA对肺癌具有相似的的术前诊断和分期作用，但对纵隔肿物的诊断效能则以纵隔镜检查术为高。

## Abstract (en)

**Background and objective:**

Endobronchial ultrasound-guided transbronchial needle aspiration (EBUS-TBNA) has been gradually universal application, but its value in the preoperative staging of lung cancer and mediastinal mass biopsy remains to be explored. The aim of this study is to evaluate the value of clinical application between mediastinoscopy and EBUS-TBNA, desiring to provide an objective basis for the rational choice applications.

**Methods:**

Between July 2009 and December 2012, mediastinoscopy patients with 361 cases accepted biopsy, including 199 cases of lung cancer and 162 cases of mediastinal mass of unknown origin, EBUS-TBNA patients with 348 cases accepted biopsy, including 216 cases of lung cancer and 132 cases of mediastinal mass. Comparing the diagnostic results and related indicators of two methods, this article analyzed the clinical value both the preoperative staging of lung cancer and the diagnosis of mediastinal mass.

**Results:**

Taking pathology diagnosis as the gold standard, the accuracy, sensitivity and specificity of mediastinoscopy and EBUS-TBNA are 98.33%, 98.17%, 100% and 90.80%, 90.00%, 100%. Two techniques in the diagnosis and staging of lung cancer have not statistically significant (*P* > 0.05), but in the diagnosis of mediastinal mass have statistical significance (*P* < 0.05).

**Conclusion:**

Mediastinoscopy and EBUS-TBNA have a similar role in the diagnosis and staging of lung cancer; but mediastinoscopy for mediastinal mass is superior to EBUS-TBNA.

纵隔镜检查术依然是肺癌术前纵隔淋巴分期的最佳手段^[1]^，支气管镜超声引导针吸活检术（endobronchial ultrasound-guided transbronchial needle aspiration, EBUS-TBNA）能否取代纵隔镜一直备受争议^[2]^。对于纵隔肿物的诊断，纵隔镜检查术的地位无可置疑，EBUS-TBNA的效能则差异较大^[3]^，大样本的对照研究较少见^[4]^，本文拟同期对照研究纵隔镜和EBUS-TBNA两种技术在肺癌诊断/分期和纵隔肿物诊断中的作用，以评估二者的应用价值和选择取向。

## 1 资料与方法

### 1.1 一般资料

2009年7月-2012年12月，上海市胸科医院共有361例患者行电视纵隔镜检查术，其中男性201例，女性160例；患者年龄18岁-81岁，平均61岁；术前确诊肺癌患者59例，其中鳞癌26例，腺癌33例，有其它恶性肿瘤病史患者18例。共行EBUS-TBNA 348例，其中男性183例，女性165例，患者年龄16岁-83岁，平均59岁；术前确诊肺癌患者41例，其中鳞癌19例，腺癌22例，有其它恶性肿瘤病史患者15例。所有患者术前均行血常规、肝肾功能、凝血功能及心电图、肺功能等相关检查化验，无手术禁忌证。住院资料以上海市胸科医院病案室存档病史资料为准。通过SPSS 13.0数据库采集以下数据：住院号、性别、年龄、诊断时年龄、手术日期、手术类型、病理类型。上述患者进一步行纵隔镜检查术或EBUS-TBNA的指征如下：①TBB或TTNA已确诊为肺癌，但纵隔淋巴结CT短径 > 1 cm或PET阳性显像，为真实评估纵隔淋巴结以确定分期^[5, 6]^；②CT或PET/CT高度怀疑肺癌并纵隔淋巴结转移但尚无明确病理，行纵隔淋巴结活检以获取病理学诊断^[7, 8]^；③病理未明的纵隔淋巴结肿大或纵隔内肿物^[9, 10]^。

### 1.2 操作过程

经颈纵隔镜检查术（standard cervical mediastinascopy, SCM）可活检的淋巴结包括：#1（颈根部和胸骨上窝）、#2（气管旁）、#3a（血管前）、#3P（气管后）、#4（气管支气管组）和#7（隆突下）。操作过程：患者仰卧位，头过度后仰伸直颈部，全麻单腔气管插管，按甲状腺手术切口消毒、铺巾，在胸骨上切迹上方约1 cm处作颈部领式切口，长约3 cm，切开皮下组织和颈阔肌，在正中线上分开两侧的颈前肌群，切开气管前筋膜至气管前间隙，用食指沿气管正中线钝性分离，形成人工隧道，沿人工隧道置入纵隔镜。重点观察区域包括第#2、#3、#4和#7组淋巴结区。胸骨旁纵隔镜（parasternal mediastinascopy, PM）可活检的淋巴结包括#5（主动脉弓下）和#6（主动脉旁）^[11]^。操作过程：在距左侧胸骨旁2 cm左右的第2或第3肋间做切口，长约3 cm，一次切开皮肤、皮下组织和肋间肌，置入纵隔镜，探查第#5组、#6组淋巴结或纵隔肿物，直视下多点活检。标本送术中冰冻或石蜡病理，若冰冻病理无法明确诊断时，重新采样送检。在保证手术安全性的前提下，尽量多处采样以保证足够的标本量和代表性，并备进一步免疫组化和基因突变检测之需。纵隔镜检查术由上海市胸科医院胸外科或上海市肺部肿瘤临床医学中心胸外科医师实施。根据IASLC 2009年颁布的肺癌区域淋巴结图谱，EUBS能够穿刺的胸部淋巴结包括部分#1（颈根部和胸骨上窝）、#2（气管旁）、#3P（气管后）、#4（气管支气管组）、#7（隆突下）和部分#10（肺门）。EBUS不能够穿刺的淋巴结为：#3a（血管前）、#5（主动脉弓下）、#6（主动脉旁）、#8（食管旁）和#9组（下肺韧带）^[12]^。EBUS具体操作过程如下：患者取仰卧位，下颌上抬，局部麻醉联合镇痛、镇静药物使用，经口插入超声支气管镜，进入气管后，根据术前胸部CT预定位置寻找穿刺目标，使其最大直径位于超声图像的中央，置入穿刺针，充盈水囊使之紧贴穿刺部位，每个目标选取不同方向穿刺3次，以获取更高的阳性率，标本送检冰冻病理，观察穿刺部位有无出血，操作过程中监测患者心率、血压及血氧饱和度。EBUS由上海市胸科医院气管镜科实施。肺癌患者中，纵隔镜检查阴性则剖胸手术，以肺部病灶及淋巴结标本获取病理学诊断；EBUS病理学阳性即终止检查，阴性者有部分再行纵隔镜检查术，或在剖胸手术时获取淋巴结的最终病理学诊断。纵隔肿物患者中，纵隔镜检查无论病理如何均为最终结论依据；EBUS-TBNA阴性者同样再行纵隔镜检查术。两者皆以术后石蜡切片病理学作为诊断金标准。

### 1.3 统计学方法

采用SPSS 13.0软件进行统计学分析。计数资料采用卡方检验，以*P* < 0.05为差异有统计学意义。计算两种技术的诊断效能，获得准确性、敏感性、特异性、阳性预测值和阴性预测值等指标。

## 2 结果

### 2.1 临床结局

共计361例患者行纵隔镜检查术，其中经颈308例，胸骨旁53例。病理阴性的39例中36例患者继行肺叶切除及系统性淋巴结清扫，3例患者改行纵隔电视胸腔镜手术（video assisted thoracic surgery, VATS）及肺门淋巴结活检。经颈纵隔镜检查术后第1天出院，胸骨旁术后放置胸管引流，术后第1天拔管，术后第2天出院。出现并发症7例，发生率1.93%，其中2例损伤喉返神经，3例损伤血管，1例伤口感染，1例损破纵隔胸膜。共计348例患者行EBUS-TBNA，60例病理阴性患者均再次行纵隔镜检查术，发现病理阳性31例，余29例病理阴性患者继实行肺癌根治术。EBUS-TBNA出现并发症4例，发生率1.15%，其中1例穿刺时出血，凝块阻塞气道，2例出现剧烈咳嗽，1例血氧饱和度下降而影响穿刺进程。

### 2.2 病理结果

共计322例纵隔镜及288例EBUS-TBNA患者获取明确病理诊断，两组获取的病理结果详见表 1。

**1:** 纵隔镜检查术和EBUS-TBNA的病理学诊断结果 Pathological diagnostic results of mediastinoscopy and EBUS-TBNA

Pathology	Mediastinoscopy	EBUS-TBNA
Lung cancer		
Squamous cell carcinoma	53	59
Adenocarcinoma	97	84
Poorly differentiated carcinoma	15	28
Small cell carcinoma	28	42
Large cell carcinoma	3	2
Carcinoid	1	1
Adenosquamous carcinoma	2	0
Mediastinal mass		
Lymphoma	16	7
Malignant thymoma (C type)	6	3
Tumors of the reproductive system	5	4
Sarcoidosis	54	31
Tuberculosis	19	11
Benign mediastinal tumor	9	5
Metastatic tumors	14	11
Negative		
Lymph node chronic inflammation and fibrous tissue proliferation	39	60
EBUS-TBNA: endobronchial ultrasound-guided transbronchial needle aspiration.		

### 2.3 诊断效能

纵隔镜检查术和EBUS-TBNA的诊断效能评价详见表 2。数据上纵隔镜略优于EBUS-TBNA，但两者之间差异无明显统计学意义（表 3）。对于肺癌，EBUS-TBNA的准确性虽然很高，但是存在一定的假阴性率，纵隔镜检查更有优势。对于小细胞肺癌，两种技术的检出率都极高，诊断率无明显差异。对结节病、结核病和淋巴瘤等纵隔肿物（其它病理因例数较少未纳入统计），EBUS-TBNA的检出率较低，存在较高的假阴性率和漏诊率，纵隔镜检查术则明显优于EBUS-TBNA，两者之间存在统计学差异。

**2:** 纵隔镜和EBUS-TBNA对肺癌及纵隔肿物的诊断效能比较 The comparison of diagnostic effect results between mediastinoscopy and EBUS-TBNA

	Mediastinoscopy			EBUS-TBNA	
	Lung cancer	Mediastinal mass		Lung cancer	Mediastinal mass
Accuracy	98.28%	98.11%		95.69%	82.64%
Sensitivity	98.03%	97.62%		94.74%	77.42%
Specificity	100%	100%		100%	100%
Positive predictive value	100%	100%		100%	100%
Negative predictive value	87.88%	91.67%		71.79%	57.14%

**3:** 纵隔镜和EBUS-TBNA对不同类型肺癌和常见纵隔肿物诊断效能比较 The comparison of diagnostic effect between mediastinoscopy and EBUS-TBNA on deferent types of lung cancer and several mediastinal masses

Pathology	Mediastinoscopy (+/-)	EBUS-TBNA (+/-)	*P*
Lung cancer	99/4	216/12	0.0711
Non-small cell lung cancer	156/3	146/11	0.0270
Small cell lung cancer	28/1	42/1	0.4879
Sarcoidosis	54/2	31/9	0.0109
Tuberculosis	19/0	11/5	0.0135
Lymphoma	16/0	7/6	0.0036

## 3 讨论

肺癌患者的生存率与病理分期密切相关。准确的病理分期不仅攸关预后判断，更是决定适宜治疗方案最重要的参考指标。美国国家综合癌症网络和美国胸科医师协会指南建议，临床Ⅲ期肺癌患者在治疗前应争取获得病理学结果以评价纵隔淋巴结转移状况^[12]^。目前，纵隔淋巴结的无创分期手段主要是CT和PET，CT的判断准确性有限，PET的假阳性率偏高，无病理学诊断成为二者的致命缺陷。纵隔淋巴结的有创分期手段包括纵隔镜检查术、EBUS-TBNA、TBNA、食管镜超声引导下针吸活检术（endoscopic ultrasound fine needle aspiration, EUS-FNA）、经胸穿刺活检术（transthoracic needle aspiration, TTNA）、胸腔镜活检术（video-assisted thoracic surgery, VATS）及常规剖胸活检术。纵隔内部结构复杂、组织来源多样，包含性命攸关的器官和组织，因而导致了纵隔病理的多样性，获取病理诊断的操作有较高的潜在风险。所以通常采取可视下或者引导下的方式获取组织标本，以尽量避免正常组织和器官的损伤。其中纵隔镜和EBUS-TBNA是目前临床上应用最为广泛的两种有创诊断技术^[13]^。

目前，纵隔镜仍然是公认的肺癌外科分期金标准^[14, 15]^，但近年有报道EBUS-TBNA有与纵隔镜相近的肺癌外科分期效果^[16, 17]^，而且EBUS-TBNA可在局麻下进行，与纵隔镜相比，并发症更少，也更加安全、方便、经济^[18]^。但EBUS-TBNA对于其它纵隔肿物的诊断价值尚无一致评价^[19]^。目前国内两种技术开展的医院尚少，前瞻性大样本的对照研究罕见于国外报道^[20]^。临床医师对两种技术的选择取向仍没有达成一致，通常根据自身的临床经验和技术状况决定采用何种方法。

EBUS-TBNA吸取的组织样本较少，部分甚至不能明确诊断，更有相当比例的病例无法明确组织分型。纵隔镜则由于能采取较多的标本而少有诊断困难。特别地，小细胞肺癌的治疗原则殊异于非小细胞肺癌，如果出现EBUS-TBNA不能分型的低分化癌，且无法排除小细胞肺癌，应当在EBUS-TBNA检查后再行纵隔镜检查术，以争取明确组织学分型。同样，如果需要足够组织标本进行*EGFR*突变检测，纵隔镜检查术同样是更可取的活检方式。

本研究中纵隔镜检查术假阴性6例，其中3例肺癌患者未检出转移淋巴结，术后病理证实均为第#7组转移，可能因为采样困难所致；2例假阴性为结节病，可能因为患者以肺门#10组淋巴结肿大为主，位于纵隔镜活检的盲区而未能诊断；另1例假阴性为小细胞癌，纵隔镜探查时见肿瘤严重侵犯纵隔血管且活检困难，少量标本未能明确诊断，虑及继续活检手术风险极大，故改行VATS活检获取病理。

EBUS-TBNA的假阴性诊断共31例，其中非小细胞肺癌9例（4.92%, 9/183），小细胞肺癌1例（2.33%, 1/43），结节病9例（22.50%, 9/40），淋巴瘤6例（46.15%, 6/13），恶性胸腺瘤1例（25.00%, 1/4），结核5例（41.67%, 5/12）。均因活检组织量过少，达不到诊断标准。可见EBUS-TBNA对于肺癌尤其是小细胞癌的诊断准确率较高，而对于其它纵隔肿物，如结节病、结核病、淋巴瘤等存在较高的假阴性率，对此类患者建议常规行纵隔镜检查术。

针对肺癌的诊断和分期，纵隔镜和EBUS-TBNA均有较高的准确性，纵隔镜略高于EBUS-TBNA，可以先采用EBUS-TBNA，对于诊断阴性者建议进一步行纵隔镜检查术；此外，若欲获得明确的肺癌病理类型诊断，或需要足够的组织标本行*EGFR*基因突变检测，应首选纵隔镜检查术。针对其它纵隔肿物的诊断，纵隔镜的准确性明显高于EBUS-TBNA，建议首选纵隔镜检查术。
